# Colonic Volvulus: An Experience at Tertiary Care Hospital in Nepal

**DOI:** 10.7759/cureus.5165

**Published:** 2019-07-18

**Authors:** Tika R Bhandari, Sudha Shahi, Rajesh Poudel

**Affiliations:** 1 Surgery, People's Dental College and Hospital, Kathmandu, NPL; 2 Otorhinolaryngology, National Academy of Medical Sciences, Kathmandu, NPL; 3 Surgery, Universal College of Medical Sciences, Bhairahawa, NPL

**Keywords:** colonic volvulus, large bowel obstruction, management, postoperative complications, volvulus belt, resource poor settings

## Abstract

Introduction: Colonic volvulus is not an uncommon cause of large bowel obstruction. Limited research has been done about colonic volvulus in our part of the world which has been regarded as "volvulus belt." The aim of this study is to evaluate the clinical features, management, and factors affecting perioperative outcomes in patients with colonic volvulus.

Methods: A retrospective review of medical records of all patients managed for colonic volvulus in Universal College of Medical Sciences, Bhairahawa from January 2012 to December 2016 was done. Data on patient demographics, clinical course, methods of treatment, and outcomes were analyzed.

Results: A total of 62 patients (46 males) were studied. Mean age was 57.9 ± 10.4 years. The most common site involved was sigmoid (85.5%). The diagnosis was made by abdominal x-rays in 39 patients (62.2%), CT scan in 13 patients (21%), and laparotomy in 10 patients (16.1%). Fifty-eight patients (93.5%) were treated surgically. Resection and ostomy was the commonest operation performed in 30 patients (48.7%) followed by resection with anastomosis in 24 patients (38.7%). The overall complication was 38.7%. There were 9.7% of deaths. In multivariate analysis, age ( ≥ 60 years) (odds ratio (OR); 27.0, confidence interval (CI); (1.92-403), P; 0.01), preoperative hypotension (systolic blood pressure <90 mmHg) (OR; 7.82, CI; (1.19-51.2), P; 0.03), and gangrenous bowel (OR; 76.7, CI (3.60-1632), P; 0.005) were significant predictors of postoperative complications .

Conclusions: Volvulus of the colon is common in males and constipation is being commonest risk factors for volvulus. Surgeons should have a high index of suspicion and should be aware of these problems to make the early diagnosis with prompt treatment and to ensure better patient outcomes in volvulus endemic areas like ours.

## Introduction

Volvulus of the colon is an axial rotation of the colon around an immobile point, generally the mesentery. Volvulus of the colon has been reported as the third common cause of large bowel obstruction in the developed world. It is followed by colorectal cancer and diverticulitis of colon [1-2]. It can affect any mobile part of the colon. Sigmoid is most commonly affected (60%-75%), followed by cecum (25%-40%), transverse colon ( 1%-4%), and splenic flexure (1%). The occurrence of colonic volvulus varies differently in different parts of the world. The endemic area also called “volvulus belt”[2], includes South America, Africa, Russia, the Middle East, Eastern Europe, Brazil, and South Asia. Out of the total intestinal obstruction cases reported in the volvulus belt, the incidence of volvulus of the colon lies between 13% and 42%. However, the incidence reported in the Western world (Western Europe, Australia, and North America) is low (less than 5%) [3-4].

Patients usually present with abdominal pain, abdominal distention, and vomiting. Delayed management can result in complications associated with closed-loop obstruction, bowel ischemia and hypovolemic shock [5] prompting for early diagnosis and management. Association of volvulus with certain conditions like pregnancy, colitis, Hirschsprung’s disease, prune belly syndrome, Chagas disease, chronic constipation, laxative abuse, freely mobile cecum, and congenital redundant colon with long mesentery have been mentioned in the literature [4,6-7]

To our knowledge, no published data is available about colonic volvulus in the context of Nepal which is a part of the volvulus belt region (South Asia). Thus, the main purpose of this study is to verify the authenticity of the name of volvulus belt given to our region. Likewise, we also aim to evaluate the clinical features, management, and factors affecting perioperative outcomes in patients with colonic volvulus. 

## Materials and methods

This is a retrospective review of the medical records of all patients who were treated for volvulus of the colon in Universal College of Medical Sciences Bhairahawa, a medical college situated in the Terai region of Nepal, from January 2012 to December 2016. The study was approved by the Institutional Review Committee of Universal College of Medical Sciences, Bhairahawa, Nepal. All patients admitted with the diagnosis of volvulus of the colon were taken into the study and diagnosis was made on the basis of clinical presentations, radiological investigations, and intraoperative findings of laparotomy. Most of our patients presented with the feature of intestinal obstruction and all patients were resuscitated with intravenous fluids, nasogastric drainage, correction of electrolyte imbalance and broad-spectrum antibiotics. Patients with the absence of features of peritonitis, underwent rectal tube decompression. However, detorsion could be achieved after the first attempt in 4 patients only.

Methods of surgical approach were chosen on the basis of hemodynamic stability of patients and intraoperative findings for individual cases. Criteria essential for primary anastomosis were considered as hemodynamic stability, good vascularity of edges, and absence of tension over the suture line after the resection of the colon. In almost all cases, peritoneal washing and placement of pelvic drain before abdominal closure was done. Perioperative data including type of surgery, intraoperative findings, postoperative complications, and length of hospital stay was recorded. Postoperative complications like surgical site infection were defined according to surgical site infection (SSI) guidelines. Likewise, intra-abdominal abscess (IAA) was defined as culture positive purulent collection. The period from the first postoperative day until discharge was defined as the length of hospital stay. All patients were followed up for 30 postoperative days.

All categorical variables were analyzed with Chi-square test or Fisher’s exact as feasible. And all continuous variables were evaluated with Mann-Whitney test or independent test, as appropriate. Postoperative complications were the outcome variables in the study. Variables that proved significant for outcome variable on univariate analysis were analyzed with multivariate analysis (logistic regression). All data were analyzed using SPSS version 25.0 for Windows. A P-value less than 0.05 was considered as statistically significant.

## Results

A total of 62 patients managed with volvulus of the colon during the study period were considered for the study. The mean age was 57.7 ± 10.4 years with a range of 14-75 years. The majority of patients were male ( 74.2%). All patients had symptoms of intestinal obstruction like abdominal distention, abdominal pain, and vomiting (late presentation). On physical examination, most of the patients were dehydrated (85%) and had associated signs of guarding (70%) and rebound tenderness (18%). Patient’s preoperative data are shown in Table 1. The diagnosis was made by abdominal x-ray in 39 patients (62.2%), CT scan in 13 patients (21%), and laparotomy in 10 patients (16.1%). The most common diagnosis was sigmoid volvulus in 53 patients (86%), followed by cecal volvulus in 9 patients (14.5%).

**Table 1:** Preoperative data Categorical variables are presented as *n* (%); Continuous variables are presented as mean. Abbreviations: ASA: American Society of Anesthesiology

Variables		No. of patients, n (%)
Age, years(mean ± SD)		57.9 ± 10.4 (Range 14-75)
Sex	Male	46 (74.2)
	Female	16 (25.8)
Preopreoperative systolic blood pressure (mmHg)	< 90	28 (45.2)
	>90	34 (54.8)
Bowel habits	Normal	25 (40.3)
	Constipation	37 (59.7)
ASA	I	37 (59.7)
	II	14 (22.6)
	II	10 (16.1)
	IV	1 (1.6)
Comorbidity	Diabetes mellites	12 (19.4)
	Cardiovascular diseases	8 (12.9)
	Hypertension	7 (11.3)
	Respiratory diseases	3 (4.8)
	Neurological diseases	2 ( 3.2)

 

We had four (6.5%) patients who underwent rectal tube decompression for sigmoid volvulus. Three out of four of them had a recurrence and they had undergone surgical treatment at subsequent presentation. Different type of approaches used during the surgery is shown in Table 2. We had complications in 24 (38.7%) patients. The most common complication was surgical site infection (12.9%) followed by lower respiratory tract infection (4.8%) as shown in (Table 2).

**Table 2:** Intraoperative data and postoperative data Categorical variables are presented as n (%); Continuous variables are presented as mean. Abbreviations: LRTI: lower respiratory tract infection, SSI: surgical site infection

Variables		No. of patients n (%)
Rectal tube reduction		4 (6.5)
Surgery	Total	58 (93.5)
	Resection anastomosis	24 (38.7)
	Resection and end-ostomy	30 (48.7)
	Reduction and pexy	4 (6.5)
Complications	Total	24 (38.7)
	SSI	8 (12.9)
	LRTI	3 (4.8)
	Stoma related problems	1 (1.6)
	Intraabdominal abscess	2 (3.2)
	Anastomatic leak	2 (3.2)
	Suture dehiscence	2 (3.2)
	Mortality	6 (9.7)
Length of Hospital stay, mean ± SD (days)		9.05 ± 4.3

We had also evaluated different factors that affect perioperative outcomes. In univariate analysis factors: age ( ≥ 60 years), sex (male), preoperative hypotension (systolic blood pressure <90 mmHg) and gangrenous bowel intraoperatively had effect on complications (P < 0.05). Among the factors discussed above in univariate analysis except for sex (male) variables like age ( ≥ 60 years), preoperative hypotension (systolic blood pressure <90 mmHg) and gangrenous bowel intraoperatively were significant predictors of postoperative complications (P < 0.05) in multivariate analysis (Table 3).

**Table 3:** Association of variables with complications Categorical variables are presented as n (%); Continuous variables are presented as mean. Abbreviations: OR: odds ratio, CI: confidence interval, ASA; American Society of Anesthesiology,  P value significant if <0.05

Variables	Complications [No. of patients, n (%)]	Univariate analysis OR,(CI) P value	Multivariate analysis OR;(CI); P value
Age( ≥ 60 years)	21(33.9)	13.4; (3.37-53.6); 0.001	27.0; (1.92-403); 0.01
Sex (Male)	14(22.6)	0.26; (0.08-0.86); 0.02	0.94;(0.007-1.25); 0.07
Preoperative hypotension	20(32.3)	18.7; (4.97-70.6); 0.001	7.82; (1.19-51.2); 0.03
Presence of comorbidity	12(19.4)	1.37; (0.49-3.84); 0.54	-
ASA ≥ III	9(14.5)	10.8; (2.08-56.0); 0.001	0.91; (0.06-13.19); 0.94
Gangrenous bowel	15 (24.2)	30.0; (5.78-155.6); 0.001	76.7; (3.60-1632); 0.005

## Discussion

To our knowledge, no data on colonic volvulus in Nepal have been published so far. Being a South Asian country, we still fall under the category of “volvulus belt.” The prevalence of colonic volvulus varies differently in different parts of the world. Volvulus of the colon is common in endemic regions than Western Europe and the United States [1]. We found colonic volvulus in 16% of all intestinal obstruction cases and specifically in 65% of total large intestinal obstruction cases in our series similarly reported from the other developing countries from Asia [8]. Mean age of our patient was 57.9 ± 10.4 and male preponderance (74.2%) in our study is comparable to the other ‘‘volvulus belt’’ countries [8]. In the Western world, elderly males ( age >70 ) are at higher risk of developing sigmoid volvulus while caecal volvulus affects younger females (age ≤ 60) which have been reported in the study by Halabi et al. [3].

The particular cause of high incidence of colonic volvulus in certain parts of the world called volvulus belt is mostly unknown. Our patients were mostly from the Terai region (southern part lowlands ) of Nepal. Most of the patients were farmers having a usual history of consumption of a heavy amount of rice and roughage including vegetables, roots and stems two times in a day or at lunch and dinner. In our patients. there were several risk factors including chronic constipation, high fiber consumption, habit of eating one large meal, old age, and diabetes. One of our adolescent patients had sigmoid volvulus with mental retardation. Volvulus in such a patient may be associated with aerophagia and constipation that induce bowel distention. Chronic constipation causes lengthening of the colon, produces a redundant sigmoid colon which is a prerequisite to volvulus. Consumption of high fiber diet is another possible factor of colonic volvulus as it causes the development of a long redundant colon. The habit of eating large meals with long gap in between the meals can also be considered for the development of sigmoid volvulus in our context.

Abdominal distension, crampy lower abdominal pain with constipation, and vomiting which is usually a late symptom are all the common presenting features of volvulus [4]. The triad is more common in endemic areas [9]. Delayed presentation is a common problem in our context. This may be due to irregular defecation habit, acceptance to pain, and abdominal distension. Similarly, the socioeconomic burden also plays a role behind the hesitation to visit the hospital for symptoms mentioned above. The problems faced by our patients are similar to those mentioned in the literature [10]. Meanwhile, in Western countries, patients are more conscious about their health and present early for help. The classic patient presentation is an institutionalized, elderly patient under psychotropic medications [11].

Diagnosis depends upon clinical presentation and radiological findings. Plain erect abdominal x-ray generally shows a dilated sigmoid colon and a coffee bean-like shape formed by grossly dilated and closely apposed sigmoid loops [12]. A whirl sign of the dilated sigmoid loop around mesocolon and a bird-beak appearance may characteristically be seen in CT abdomen [13-14]. CT scan has high diagnostic utility and is therefore highly recommended. However, the radiological findings seen in x-rays were diagnostic in most of our patients with sigmoid volvulus. Moreover, since most of our patients were from the low socioeconomic background, we considered x-rays as a diagnostic tool. However, in patients where plain x-rays abdomen couldn’t yield significant diagnostic value, a CT scan was performed.

The main aim of the treatment is to release the bowel obstruction, prevent ischemia and gangrene, and prevent recurrence [15]. Selection of the most suitable therapeutic methods depends on the clinical status of the patient, the location of the problem, the suspicion or presence of peritonitis, bowel viability, and on the experience of the surgical team. The accessibility of recent advanced techniques in resource-poor hospitals and its relevance is still challenging. Thus, the reduction of a sigmoid volvulus by proctoscopy and rectal tube decompression are common non-operative measures that we generally practice in our center. Contraindication of the procedures above are features of perforation. Reduction by colonoscopy or sigmoidoscopy could be achieved only in 40%-60% cases mentioned in the literature [16-17]. However good results can be obtained with a flexible scope where the success rate varies from 70 to 92.9% [18]. The recurrence rate of 35%-90% with non-operative therapy has been reported [19-20]. Thus, surgical management has been considered by most surgeons. However, for cecal volvulus, such non-operative procedure has not been found beneficial.

During the surgical treatment, individual risk factors and operative findings are the main factors for determining the choice of operation. There are various surgical options which include derotation alone, derotation with colopexy, resection with primary anastomosis, and resection with end-ostomy. In our case, we performed exploratory laparotomy and resection of a redundant colon with colocolic end to end anastomosis and resection and end ostomy with no recurrence rate. The findings are similar to those reported in most of the literature. A minimally invasive laparoscopic surgery has been recommended as one of the treatment options of volvulus with considerable outcomes [20-21]. Due to lack of expertise and infrastructure, we could not perform laparoscopic surgery in our set up.

We had 38.7% postoperative complications which are comparable to other studies. Surgical site infection was the most common complication followed by lower respiratory tract infection comparable to another study [22]. Anastomotic leak was found in two of our patients (3.2%). One out of two patients with anastomotic leak resolved spontaneously with conservative management but one patient died due to sepsis. Similar reports of the anastomotic leak have been mentioned in the literature [8,23]. We had a mortality of 6 (9.7%) patients. Other studies also had similar reports on mortality [13,24]. We also evaluated different factors that affected perioperative outcomes. Age ( ≥ 60 years), preoperative hypotension (systolic blood pressure <90 mmHg) and gangrenous bowel intraoperatively had a significant effect on complications (P< 0.05) in our study. Traore et al. in their study reported that therapeutic approach and general condition of the patient had a significant effect on complication among those with age greater than 60, the therapeutic approach practiced the general condition of the patient, duration of the surgery and comorbidity [25]. Bhatnagar et al. in their study also reported aging, the presence of shock, and general condition of the patient as independent risk factors associated with postoperative complications [26].

Poor access to healthcare makes it more challenging for early diagnosis and management. Despite all, we believe that this is the first study in the context of colonic volvulus and this shall definitely add to the data on “volvulus belt.” The limitations of our study were; a small study population, retrospective and single centered design. Hence, in order to validate our findings, further, appropriately designed prospective studies are recommended.

## Conclusions

Volvulus of the colon is common in males and constipation is being the commonest risk factor for volvulus. Surgeons should have a high index of suspicion and should be aware of these problems to make an early diagnosis with prompt treatment and to ensure better patient outcomes by preventing morbidity and mortality mainly in volvulus endemic areas like ours.
